# The Impact of Chronic Pain, Stiffness and Difficulties in Performing Daily Activities on the Quality of Life of Older Patients with Knee Osteoarthritis

**DOI:** 10.3390/ijerph192416815

**Published:** 2022-12-14

**Authors:** Agata Wojcieszek, Anna Kurowska, Anna Majda, Henryk Liszka, Artur Gądek

**Affiliations:** 1 Institute of Nursing and Midwifery, Faculty of Health Sciences, Jagiellonian University Medical College, 30-126 Krakow, Poland; 2 Trauma and Orthopaedics Clinical Department, University Hospital, Jagiellonian University Medical College, 30-688 Krakow, Poland

**Keywords:** osteoarthritis, quality of life, disability, gonarthrosis, old age, knee joint, pain

## Abstract

Osteoarthritis causes a number of physical ailments, which result in the deterioration of a persons’ general health and reduction of their ability to move freely. This cross-sectional study was designed to assess the impact of physical ailments in the course of knee osteoarthritis (KOA) on the quality of life (QoL) of patients in early old age. An anonymous survey was conducted by the use of the recognized research tools: Western Ontario scale and McMaster Osteoarthritis Index (WOMAC), The Index of Severity for Knee Disease (ISK) and World Health Organization Quality of Life—BEFF (WHOQOL-BREF). The study involved 300 people aged between 60 and 75 years old, including 150 patients diagnosed with gonarthrosis and 150 people without lower limb complaints. The significant intensification of the symptoms of knee osteoarthritis was associated with a worse assessment of health (*p* < 0.001), overall quality of life (*p* < 0.001) and in the following domains: physical (*p* < 0.001), mental (*p* < 0.001) and environmental (*p* < 0.001) in a group of patients with KOA. These findings suggest that taking measures to reduce knee pain and improve function may have an impact on improving the overall quality of the life of people in their early old age.

## 1. Introduction

Osteoarthritis (OA) is the most common chronic disease of the musculoskeletal system. The definition of osteoarthritis has evolved over the past two decades and is now viewed as a complex of clinical, pathophysiological, biochemical and biomechanical changes with many complex etiologies [1]. Disease processes affect not only the articular cartilage, but respond to the whole joint, including the subchondral layer of bones, ligaments, joint capsule, synovium and periarticular muscles. These changes can be initiated by many factors: genetic, biological, metabolic or traumatic [2]. Estimates of the World Health Organization (WHO) show that 9.6% of men and 18% of women over the age of 60 have symptomatic osteoarthritis. Additionally, this disease is considered to be the fourth cause of global disability [3,4]. The disease process may affect the hip, knee, ankle, shoulder, elbow, wrist, interphalangeal, metacarpophalangeal or spine joints. Among all these forms, it is the knee osteoarthritis, also called gonarthrosis, that is the most troublesome for patients [5].

The dominant symptom of osteoarthritis is pain, which is perceived as the result of complex interactions between biological and psychosocial factors [6]. Pain and discomfort associated with it usually occurs when moving or loading the joint. It can vary in severity, from mild to very severe. As the disease progresses and the damage to the joint structure increases, pain may also appear at rest. Sleep disturbances that interfere with the regeneration of the body can further aggravate the pain through the associated fatigue and lack of well-being. Other symptoms and signs that may occur in patients with OA include: crackles, reduced range of motion, effusion, abnormal joint positioning and weakness of the surrounding muscles and gait abnormality [7].

The natural course of pain and the degree of physical functioning in a group of people with knee osteoarthritis is highly individual and variable, as the factors limiting physical activity differ, depending on the severity and extent of the degenerative changes in the knee [8,9]. All the perceived physical ailments limit the patient’s activity, which may determine, e.g., cognitive deterioration [10,11], physical disability [12], tumbles [13], poor mental state [14,15] and death [16].

The Quality of life (QoL) is a concept that has become the subject of numerous scientific studies aimed at exploring many areas of an individual’s life. In 1994, WHO established the World Health Organization Quality of Life (WHOQOL) section, which defined quality of life as an individual perception of one’s life situation in a broad context, taking into account cultural conditions, individual expectations and social norms [17]. Measurements of QoL may be general or disease-specific. Overall QoL measures evaluate the perceptions of physical, mental, social and environmental health that are relevant to different people. Despite different concepts and perceptions of overall QoL, researchers agree on several issues regarding this construct: overall QoL is subject to subjective assessment, consists of four basic areas of the functioning of the individual, it is a process that changes over time and is susceptible to the influence of additional internal and external factors [18,19]. In turn, specific measures of QoL include characteristic symptoms associated with the disease, activity limitations and the impact of the disease on daily functioning. The exploration of large databases on the health-related quality of life (HRQoL) has demonstrated that people with a very similar health status and suffering from the same disease can radically differ in terms of their quality of life, due to different expectations and behavior towards the disease and people’s ability to deal with it [20].

There is still no gold standard in the diagnosis of quality of life deficiency in clinical management in Poland. This problem is focused especially in the areas of quality of life based on the psychophysical condition and relieving chronic pain in orthopedics, where the extensive joint pathology generates a significant increase in the pain that affects the quality of life and the efficiency of vital systems. In the field of interventional medicine, there are still few researchers who deal with this problem scientifically, and in clinical medicine, research on the genesis of pain, physical and mental condition and quality of life is not often undertaken by doctors and other healthcare professionals.

The aim of the study was to assess the impact of symptoms occurring in the course of knee osteoarthritis (KOA), such as: joint pain and stiffness, as well as functional limitations, on the quality of the life of patients in early old age (according to the WHO periodization of old age) [21]. At the initial stage of designing the study, the research team made an assumption that the severity of KOA symptoms may affect the quality of life of the patients, and also in the psychological, social and environmental domains. We recognized that the inclusion of a comparison group in the study would be valuable because of the possibility of specifying differences in quality of life and their domains among people with and without symptoms of KOA.

## 2. Materials and Methods 

### 2.1. Research Design and Data Collection 

The study was cross-sectional and was carried out with the diagnostic survey method. The technique that was used to collect the material for the research was the survey technique and the scaling technique.

An anonymous study in the group of patients with KOA (study group—SG) was conducted from January 2019 to February 2020 in three Krakow hospitals located in the Lesser Poland Voivodeship—in an orthopedic ward and an orthopedic clinic. The study was preceded by the consent of the management of the institutions where the study was conducted. A research team member obtained information from a KOA patient about the purpose of the visit to the clinic or ward, presented information about the study (topic, purpose, study inclusion and exclusion criteria, etc.) and offered to participate in an anonymous survey. The participants were informed that the study was voluntary, anonymous and it is possible to withdraw at any stage of the study without consequences and impact on the further treatment process in medical facilities. A patient who agreed to the study and met the inclusion criteria in the study, supplemented the prepared research tools. The information obtained from the patient was not verified by access to the documentation.

The research in the comparative group (CG) was carried out in senior clubs and day care homes for the elderly in the period from May 2019 to November 2020 in the Lesser Poland Voivodeship. Respondents were provided with information about the subject, purpose of the study and criteria for inclusion in the study, as well as the possibility of withdrawing from the participation without any consequences.

The survey consisted of one stage—filling in the questionnaire by the respondent. The selection of participants for the study was deliberate—the study included people diagnosed with gonarthrosis and people without osteoarthritis of the knee joint.

### 2.2. Instruments

(a) The Western Ontario and McMaster Osteoarthritis Index Scale (WOMAC)

The Western Ontario and McMaster Osteoarthritis Index Scale serves as a recognized, multi-dimensional tool for assessing and self-assessing the level of disability in patients with osteoarthritis of the knee and hip joints. The tool consists of 24 questions with a 5-point adjectival answer scale. The results are obtained for each of the three components, e.g., pain from 0 to 20 points, stiffness from 0 to 8 points, functional activity from 0 to 68 points, which together give a global score from 0 to 96 points, which can be converted to a percentage value. The higher the score, the higher the level of disability in the joints of the lower extremities [22,23,24].

(b) The Index of Severity for Knee Disease (The Lequesne Index; ISK)

The Lequesne Index is a questionnaire for assessing the dysfunction of the knee and hip joints. The Polish version of the questionnaire was made available by Dr. Nonna Anna Nowak from the Center for Osteoporosis and Osteoarticular Diseases in Bialystok [22]. The questionnaire consists of 10 questions. Five of them concern the perception of pain when walking, standing, getting up from a sitting position, night rest, and morning discomfort. In each question, the respondent can obtain from 0 to 2 points. One of these questions relates to the maximum distance covered and is rated on a scale of 0 to 6 points, depending on the declared distance traveled. The next four questions relate to activities related to everyday life and are scored from 0 to 2 points. The overall test score ranges from 0 to 24 points. The higher their number, the higher the degree of lower limb function impairment. The Lequesne pain-functional index has a satisfactory internal consistency due to the Cronbach’s alpha coefficient, which ranges between 0.74–0.80 for osteoarthritis of the knee joint [25,26].

Based on the test result, each subject is classified into one of the following categories:

– 0 points: no functional joint disorders (lack);
– Points 1 to 4: gentle;
– Points 5 to 7: moderate;
– Points 8 to 10: severe;
– Points 11 to 13: very severe;
– Above 14 points: extremely.

(c) The World Health Organization Quality of Life Scale (WHOQOL-BREF)

The shortened Questionnaire for Life Quality Assessment was created on the basis of the WHOQOL-100 questionnaire and is a universal research tool for assessing the quality of life of healthy and sick people. The Polish version of the tool was developed by Jaracz K. and Wołowicka L. from Poznan University of Medical Sciences [25]. It contains 26 questions analyzing four areas of life: physical (questions number: 3, 4, 10, 15, 16, 17, 18), psychological (questions number: 5, 6, 7, 11, 19, 26), social (questions no: 20, 21, 22) and environmental (questions number: 8, 9, 12, 13, 14, 23, 24, 25). The questionnaire also includes two separate questions concerning the individual perception of the quality of life and one’s own health. Scoring ranges from 1 to 5 and has a positive direction. WHOQOL-BREF demonstrated acceptable internal consistency for all domains (Cronbach’s alpha coefficient ranged from 0.76 to 0.84) [27,28]. WHO consent was obtained to use the WHOQOL-BREF scale in this study; authorization number: 371022.

(d) Self-Made Questionnaire

Important sociodemographic data were collected, such as: age, sex, place of residence, declared duration of illness and the type of work performed in the past. One of the questions in the questionnaire referred to anthropometric data (weight, height) to calculate the body mass index (BMI).

### 2.3. Participants

The study included 174 people in early old age, i.e., between 60 and 74 years of age with diagnosed osteoarthritis of the knee, and 170 people in early old age, e.g., between 60 and 74 years of age without a KOA diagnosis. At the stage of analyzing raw results, the research team decided to exclude from the analysis 44 questionnaires that had significant deficiencies in the respondents, e.g., no answers to questions, incomplete sociodemographic data. The analysis included 300 sheets of the research tool. The questionnaire return rate was 87.20%.

The inclusion and exclusion criteria for SG and CG are presented below (see Table 1).

### 2.4. Data Analysis 

The analysis of quantitative variables was performed by calculating the mean, standard deviation, median, quartiles, minimum and maximum. The analysis of qualitative variables was performed by calculating the number and percentage of occurrences of each value. 

The comparison of the values of the qualitative variables in the groups was performed using the Chi-square test or the Fisher’s exact test (when the expected values were less than 5). The comparison of the values of quantitative variables in two groups was performed using the Mann-Whitney U test. The correlations between quantitative variables were analyzed using the Spearman correlation coefficient. A significance level of *p* < 0.05 was adopted in all the tests performed. The research results were prepared using the statistical package R 4.0.1 [29].

### 2.5. Ethical Considerations 

Before starting the research, on 25 January 2018, the consent of the Bioethics Committee of the Jagiellonian University (KBET/1072.6120.1.2018) was obtained. The study was designed, conducted, and its results developed in accordance with the principles of Good Scientific Practice and The Helsinki Declaration.

## 3. Results

Both groups were dominated by women (54.67%). The mean age was 67 ± 4 years for the SG and 66 ± 3 years for CG. Just over half of the respondents (55.33%) lived in rural areas. The highest percentage of indications concerned people who had previously performed mental work (40.00%—the study group; 46.00%—the comparative group) and those who had a body mass index within the normal range (51.33%—the study group; 62.00%—the comparative group). In the group of patients with OA of the knee, the majority of patients rated the duration of OA of the knee from at least one year to 5 years (42.00%) (see Table 2).

When comparing the two groups (SG, CG), it can be noticed that they differed significantly in terms of age (*p* = 0.035) and the type of work performed in the past (*p* = 0.036). The respondents from the study group were older and performed physical work significantly more often. The groups participating in the study did not differ significantly in terms of sex, place of residence or BMI (Body mass index) (*p* > 0.05) (see Table 2).

The group of patients with osteoarthritis of the knee joint was dominated by the groups of patients with severe (36.67%) and very severe (34.67%) lower limb joint function impairment (see Table 3).

The dominant percentage of the surveyed patients with KOA reported moderate intensity of pain during: walking on a flat surface (N = 90; 60.00%), lying in bed at night (N = 86; 57.33%) and at rest (N = 64; 42.67%). In the case of knee loading (N = 61; 40.67%) and walking up the stairs (N = 61; 40.67%), the majority of respondents assessed this pain as severe. Regarding joint stiffness, for the majority of people with KOA, morning stiffness was moderate (N = 107; 71.33%) and evening stiffness was rated as severe (N = 65; 43.33%). The analysis of difficulties in performing walking activities demonstrated that activities such as: going down stairs (N = 107; 71.33%), getting up from a sitting position (N = 73; 48.67%), standing (N = 70; 46.67%), bending to the floor (N = 75; 50%), walking on a flat surface (N = 70; 46.67%), getting in and out of the car (N = 74; 49.33), doing shopping (N = 52; 34.67%), staying in bed (N = 46; 30.67%), taking off socks (N = 58; 38.67%), getting out of bed (N = 75; 50%), sitting down (N = 68; 45.33%) and light housework (N = 62; 41.33%) (N = 78; 52%) were characterized by moderate difficulty in the opinion of the majority of respondents. On the other hand, putting on socks (N = 65; 43.33%), getting in and out of the bath/shower (N = 67; 44.67%), sitting down and getting up from the toilet (N = 75; 50%), heavy housework (N = 62; 41.33%) and walking up the stairs (N = 73; 48,67%) were performed with great difficulty in the opinion of the majority surveyed (see Table 4).

Overall scores for disability (56.63%; SD = 12.18), pain (52.93%; SD = 14.71), stiffness (53.67%; SD = 14.67) and physical activity 58.07%; SD = 11.72) were above the middle value. The points possible to gain ranged from 10.00% to 85.00% (see Table 5).

Almost every second person with gonarthrosis assessed their quality of life as “neither poor nor good” (49.33%), and every fourth respondent considered it “good” (26.67%). In the CG, half of the respondents indicated a good quality of life (50.67%), and every fourth respondent indicated “neither poor nor good” (27.33%) (see Table 6).

More than half of the respondents from SG (60.00%) and the CG (52.00%) assessed their health as “neither satisfied nor dissatisfied”. In turn, every fourth respondent with KOA (26.00%) and every fifth without gonarthrosis considered it “dissatisfied” (20.67%) (see Table 6).

People from the SG and CG rated their quality of life the highest in the domain of psychological domain and the worst in the physical domain (see Table 6).

Perception of own health (*p* = 0.012) and quality of life (*p* = 0.001), taking into account the physical (*p* < 0.001), mental (*p* < 0.001), social (*p* = 0.024) and environmental (*p* < 0.001) domains, were significantly better in the CG than in the SG (see Table 7).

The degree of the impairment of joint function of lower limbs, the level of disability, including the subscales concerning pain, stiffness and functional activity, correlates significantly (*p* ˂ 0.05) and negatively (r ˂ 0) with the perception of the quality of life and one’s own health, and with the quality of life in the areas of physical, psychological and environmental domains (see Table 8).

Age correlated significantly (*p* ˂ 0.001) and positively (r ˃ 0) with functional limitations in each of the studied areas, so the older the age, the higher the level of disability of lower limbs (including subscales) and the higher the degree of impairment of their functions. Overall disability of the lower limbs (*p* < 0.001), including the subscales for pain (*p* < 0.001), stiffness (*p* = 0.006), physical activity (*p* < 0.001) and the degree of joint function impairment (*p* < 0.001) were significantly higher in men than in women. The level of lower limb disability and the degree of impairment of their functions were significantly greater in the overweight or obese group (*p* < 0.001).

## 4. Discussion 

The conducted research was aimed at checking whether the intensity of pain, stiffness and impairment of daily activities differentiated the quality of life of patients with diagnosed osteoarthritis of the knee joint and without the diagnosis of this disease. The use of generic and specific tools at work made it possible to obtain comprehensive assessment of the quality of life, where the physical condition of the patient was the main point of reference.

Knee pain is common, and the associated reduction in physical performance is a strong predictor of future disability. Analyzing the results of the WOMAC scale and the ISK questionnaire, it was found that patients with gonarthrosis experienced a number of physical ailments, which limited the functioning of the lower limbs’ possibilities. It is worth mentioning, however, that nearly half of the respondents indicated a relatively short duration of the disease, e.g., from 1 to 5 years, so one may wonder if this is due to the underestimation or rapid progression of the disease. Nevertheless, every second respondent had an abnormal body weight. In her study, M. Sierakowska et al. attempted to assess potential health problems resulting from osteoarthritis with the help of, inter alia, the WOMAC scale and the ISK questionnaire. A total of 100 patients participated in these studies, including 41 people with gonarthrosis. The values obtained in the scope of the aforementioned research tools were lower. However, M. Sierakowka qualified younger people for her research, i.e., those over 45 years of age. The authors emphasized the progression of degenerative changes dependent on the duration of the disease and, consequently, a significant deterioration in the physical functioning of patients, which was also reflected in the authors’ own research [30]. Moreover, lower WOMAC scores were obtained in the Italian [31] and Egyptian studies [32]. 

In our study, knee joint loading generating more pain and stiffness was greater at the end of the day. An additional analysis of the respondents’ answers in terms of the WOMAC scale made it possible to identify those activities whose performance causes more difficulties. The examined people with KOA reported great difficulties while using the bath and shower, using the toilet and putting on socks and heavy housework. In other publications, patients most often reported problems with running, heavy housework, getting in and out of the bath [33], kneeling [34] and squatting [35]. Moreover, walking up the stairs is a typical everyday activity, which is associated with the occurrence of great discomfort, which was also reflected in our own research [9,36]. Analyzing the results of our own research, it can be noticed that the greater severity of knee complaints was determined by: male sex, older age and higher BMI. A number of publications confirm that higher body weight and hard physical work may determine more severe course of a degenerative disease. The only controversy is the fact that men had a higher level of disability of the lower limbs and greater functional limitations than women, and the literature often postulates that a situation is quite different [37]. 

This manuscript shows a significant dissimilarity in the perception of quality of life (within each domain) and one’s own health between SG and CG. People without lower limb complaints obtained better results on the WHOQOL-BREF scale than people with gonarthrosis, which is in line with the original assumption of the study. The average assessment of the quality of life in SG was 3.21 points, and although such a result is between the answers: “neither bad nor good” and “good”, the advantage of the intermediate variant should be noticed here, which may indicate ambivalent feelings towards the current situation of the patient (the questions included in the scale refer to the last 4 weeks). In the case of their perception of their own health, the study group felt much worse, oscillating between dissatisfaction and the intermediate variant, i.e., “neither satisfied nor dissatisfied” (2.88 points). When analyzing the distribution of responses in SG, a significant percentage of responses showing dissatisfaction with one’s health condition can be noticed, with a relatively small number of people declaring satisfaction. Health self-assessment is considered to be a relatively good measure of health and also an independent predictor of mortality [38]. In terms of domains, patients with OA scored the worst in the physical domain of QoL, which consisted of the following aspects: daily activities, addiction to drugs and auxiliaries, energy and fatigue, mobility, pain and discomfort, sleep and rest and ability to work. Each of the aforementioned areas is disturbed in the case of people with KOA, which was already mentioned in the introduction to this study. A study conducted in 2017 on a group of 100 people with KOA gave similar results to those obtained in their own research. In the study by A. Czykiety et al., the average assessment of the quality of life was 3.56 points, which means that the respondents assessed their quality of life between neutral (“neither good nor bad”) and good. The subjective health condition was between unsatisfactory and average (2.8 points). Slightly different results were obtained in terms of the quality of life domains, because in the cited study, the social domain was rated the highest, followed by the physical domain, and the environmental domain as the lowest [39].

Osteoarthritis is manifested by pain, stiffness and daily activity deficits, which is directly reflected in the results obtained in the general quality of life questionnaires [40] and in those specific tools that examine the above-mentioned parameter in relation to health [38,39]. In the course of the research procedure, it was demonstrated that the physical condition of the lower limbs influenced the general perception of the quality of life of the subjects. In other words, the progressive nature of the disease was conducive to increasing dissatisfaction with one’s health, mental well-being and social performance. The statistical analysis did not demonstrate any relationship between the physical state and the social domain. Moreover, pain, stiffness and physical abilities of the patients were significantly and negatively correlated with their quality of life, health and in the physical, psychological and social domains. Thus, these features largely mediate the relationship between gonarthrosis and quality of life. Similar results were obtained in a study conducted among 100 people with osteoarthritis of the knee joint. They demonstrated that, inter alia, pain intensity influenced the quality of life and health perception as well as the psychological, social and environmental domains of quality of life. These correlations are negative, which means that the greater the pain intensity, the lower the quality of life in these areas. An interesting comparative study was conducted among 264 patients with osteoarthritis of lower limb extremities (hip and knee) and 112 healthy people. The QoL was measured with the SF-36 questionnaire. The authors demonstrated a negative impact of OA on the quality of life of the respondents, especially in terms of the physical and psychological domains, compared to healthy subjects [41]. Subsequent studies were conducted by Jaiswal et al. on a group of 454 elderly people, 292 of whom were diagnosed with KOA. The aim of these studies was to determine the prevalence of knee OA and to assess QoL based on the WHOQOL-BREF questionnaire. Researchers demonstrated a significant difference in the quality of life of the elderly suffering from knee OA than those who were not suffering from it. This difference was significant in all of the domains of quality of life, but the maximum effect of knee OA was on the physical and psychological domain [42]. Similar results were obtained in our own study. The impact of chronic pain on the physical domain of the quality of life is obvious, but its impact on the mental layer is much more complex. When analyzing scientific articles devoted to this subject, it can be concluded that there is a mutual dependence between the severity of pain ailments and the mental state. On the one hand, pain in osteoarthritis may affect the mood and well-being of an individual [43]. On the other hand, the persistent negative cognitive style, characterized by helplessness, a tendency to exaggerate and reflective thoughts about one’s own pain, determines its increased perception [44]. Among other factors that play an intermediate role between the perceived pain and the perception of the quality of life among KOA patients, there are, for example, depressive disorders [45], worse sleep quality [46] or loneliness [47]. In our study, we focused on patients with KOA, but there is also a worse assessment of the quality of life in patients suffering from other diseases related to the osteoarticular system, e.g., rheumatoid arthritis (RA) [48] or osteoporosis [49] compared to a relatively healthy control group. In their cross-sectional study, Siboni et al. examined 625 chronically ill patients. Results of the present work demonstrated that affliction with chronic diseases can affect QoL of the individuals [50]. 

The cited research confirms the legitimacy of designing and implementing new solutions that may contribute to the improvement of QoL in people with KOA. Our study confirmed that physical health is an important determinant of the quality of life; therefore, any negative changes in health will significantly modify the quality of life. First of all, steps should be taken to increase patients’ awareness of the key determinants of pain and to learn about the range of therapeutic interventions that can reduce or relieve symptoms. It is especially important to promote non-pharmacological activities in osteoarthritis, e.g., increasing physical activity or weight reduction. Pharmacological treatment, although it is considered effective, may cause side effects in the form of gastrointestinal disorders and multi-organ failure in the long run [51].

There are several limitations to this study. The first is related to the cross-sectional design itself, which makes it impossible to assess cause-effect relationships. During the course of the study, no data on the incidence of comorbidities in the studied groups of patients and no information on the treatment used were obtained, which seems to be of great importance for many indicators assessed in this study. Moreover, on the basis of the WOMAC scale result, it is impossible to adjectively define the level of disability of the lower limbs, as it does not provide such possibilities. There are also problems in reporting WOMAC scores due to the lack of clarity and transparency in the way this tool is used in clinical trials, which made it difficult to relate one’s own results to those of other researchers [52].

## 5. Conclusions

- The conducted study demonstrated that the perception of one’s own health and quality of life, taking into account each of the domains, was significantly better in the group of people with undisturbed motor skills. Analyzing the results in the group of people with gonarthrosis, it was noticed that along with the increase in the impairment of the functions of the lower limb joints and the severity of KOA symptoms, the respondents were assessed as worse in their health, overall quality of life and quality of life in the physical, psychological and environmental domains.
- Due to the fact that the genesis for many crises in old age is the deterioration of the physical condition, it seems necessary to further diagnose the sense of quality of life in this group, which may turn out to be one of the forms of secondary prevention of health problems occurring in old age.

## Figures and Tables

**Table 1:** Inclusion and exclusion criteria for SG and CG.

Inclusion Criteria	Exclusion Criteria
Study group (SG)	
– Women and men in between 60 and 75 years of age – Consent to participate in the study – Diagnosis towards KOA – Clinical manifestation of KOA by the presence of at least one of the following symptoms: knee pain, stiffness, and impairment of the patient’s physical activity – Lack of surgical intervention in the lower limbs in the year preceding the study – Patient’s statement about the lack of diagnosis of a mental illness	– Women and men under the age of 60 and over 75 – No consent to participate in the study – No diagnosis for KOA – Asymptomatic form of KOA – Performing a surgical intervention in the year preceding the examination in the area of lower limbs – Patient’s declaration of mental illness
Comperative group (CG)	
– Women and men in between 60 and 75 years of age – Consent to participate in the study – No diagnosis for KOA – Absence of lower limb complaints: pain, stiffness and limitation of the patient’s physical activity – Maintained mobility within lower limbs – Lack of surgical intervention in lower limbs in the year preceding the study – Patient’s statement about the lack of diagnosis of a mental illness	– Women and men under the age of 60 and over 75 – No consent to participate in the study – Diagnosis towards KOA – Presence of at least one of the following symptoms: lower limb complaints: knee pain, stiffness, impaired patient activity – Impaired motor skills in lower limbs – Performing a surgical intervention in the year preceding the examination in the area of lower limbs – Patient’s declaration of mental illness

**Table 2:** Comparison of the study participants in terms of gender, age, place of residence and type of job performed in the past and BMI.

Variables		Group		*p*
		SG n (%)	CG n (%)	
Age (years)	M ± SD	67 ± 4	66 ± 3	*p* = 0.035 *
	Me	67	65.5	
	Q1–Q3	63–70	63–68	
Gender	Female	82 (54.67)	82 (54.67)	*p* = 1
	Male	68 (45.33)	68 (45.33)	
Place of residence	Rural	83 (55.33)	70 (46.67)	*p* = 0.166 **
	Urban	67 (44.67)	80 (53.33)	
Type of work performed in the past	Physical work	48 (32.00)	35 (23.33)	*p* = 0.036 ***
	Mental work	60 (40.00)	70 (46.67)	
	Both	41 (27.33)	37 (24.67)	
	Not applicable	1 (0.67)	8 (5.33)	
BMI	Norm	77 (51.33)	93 (62.00)	*p* = 0.113 ***
	Overweight	69 (46.00)	56 (37.33)	
	Obesity	4 (2.67)	1 (0.67)	
Declared duration of the disease	Up to one year	11 (7.33)	-	-
	1–5 years	63 (42.00)	-	
	6–10 years	39 (26.00)	-	
	11–15 years	30 (20.00)	-	
	Over 15 years	7 (4.67)	-	

M—mean, SD—standard deviation, Me—median, Q1—quart 1, Q3—quart 3 and *p*—statistical significance level. * The Mann-Whitney U Test; ** Chi Square test; *** Fisher’s exact test.

**Table 3:** The degree of functional limitation in group of patients with gonarthrosis.

ISK Number of Points	Interpretation—Impairment of Joint Function	n	%
0	Lack	0	0.00
1–4	Gentle	0	0.00
5–7	Moderate	5	3.33
8–10	Severe	55	36.67
11–13	Very severe	52	34.67
14–24	Extremely severe	38	25.33

Source: authors’ analysis.

**Table 4:** Distribution of respondents’ responses in terms of pain, stiffness and limitations in daily activity.

SG										
**The WOMAC Scale** **The Occurrence of Pain Complaints during:**	**Points**									
	**None**		**Slight**		**Moderate**		**Large**		**Extreme**	
	**N**	**%**	**N**	**%**	**N**	**%**	**N**	**%**	**N**	**%**
1. Walking on a flat surface	0	0.00	14	9.33	90	60.00	33	22.00	13	8.67
2. Walking up the stairs	0	0.00	20	13.33	56	37.33	61	40.67	13	8.67
3. Lying in bed at night	5	3.33	43	28.67	86	57.33	16	10.67	0	0.00
4. Rest (sitting, lying down)	5	3.33	47	31.33	64	42.67	34	22.67	0	0.00
5. Lower limb loads	1	0.67	26	17.33	58	38.67	61	40.67	4	2.67
**The WOMAC Scale** **Occurrence of Stiffness during:**	**Points**									
	**None**		**Slight**		**Moderate**		**Large**		**Extreme**	
	**N**	**%**	**N**	**%**	**N**	**%**	**N**	**%**	**N**	**%**
6. Morning stiffness	0	0.00	13	8.67	107	71.33	30	20.00	0	0.00
7. Stiffness later in the day	1	0.67	36	24.00	48	32.00	65	43.33	0	0.00
**The WOMAC Scale** **Difficulties in Performing Daily Activities**	**Points**									
	**None**		**Slight**		**Moderate**		**Large**		**Extreme**	
	**N**	**%**	**N**	**%**	**N**	**%**	**N**	**%**	**N**	**%**
8. Going down the stairs	0	0.00	8	5.33	107	71.33	34	22.67	1	0.67
9. Walking up the stairs	0	0.00	5	3.33	59	39.33	73	48.67	13	8.67
10. Getting up from a sitting position	1	0.67	20	13.33	73	48.67	39	26.00	17	11.33
11. Standing	4	2.67	49	32.67	70	46.67	27	18.00	0	0.00
12. Bend down to the floor	0	0.00	8	5.33	75	50.00	46	30.67	21	14.00
13. Walk on a flat surface	4	2.67	20	13.33	70	46.67	53	35.33	3	2.00
14. Getting in/out of the car	1	0.67	8	5.33	74	49.33	47	31.33	20	13.33
15. Going shopping	1	0.67	34	22.67	52	34.67	50	33.33	13	8.67
16. Putting on socks	1	0.67	16	10.67	65	43.33	65	43.33	3	2.00
17. Lying in bed	5	3.33	37	24.67	46	30.67	39	26.00	23	15.33
18. Taking off your socks	2	1.33	19	12.67	58	38.67	50	33.33	21	14.00
19. Getting out of bed	1	0.67	21	14	75	50.00	50	33.33	3	2.00
20. Getting in or out of the bath	0	0.00	23	15.33	33	22.00	67	44.67	27	18.00
21. Seat	0	0.00	38	25.33	68	45.33	44	29.33	0	0.00
22. Getting up/sit down from/on the toilet	1	0.67	6	4.00	67	44.67	75	50.00	1	0.67
23. Heavy housework	0	0.00	22	14.67	56	37.33	62	41.33	10	6.67
24. Light housework	0	0.00	13	8.67	78	52.00	59	39.33	0	0.00

**Table 5:** The results of the assessment of pain, stiffness, functional activity and the level of disability in group of patients with gonarthrosis.

WOMAC Categories	M	SD	Me	Min	Max	Q1	Q3
Pain	52.93	14.71	55.00	10.00	85.00	40.00	60.00
Stiffness	53.67	14.67	50.00	12.50	75.00	50.00	62.50
Physical activity	58.07	11.72	58.09	17.65	77.94	50.00	64.71
Total score	56.63	12.18	57.29	16.67	79.17	48.96	62.50

M—mean, SD—standard deviation, Me—median, Min—minimum, Max—maximum, Q1—quartile 1 and Q3—quartile 3. Source: authors’ analysis.

**Table 6:** Diversification of SG and CG in terms of quality of life outcomes.

**The Perception of the Quality of Life** **WHOQOL-BREF**		**SG**		**CG**		**Total**	
		**n**	**%**	**n**	**%**	**n**	**%**
Very poor		0	0.00	0	0.00	0	0.00
Poor		27	18.00	20	13.33	47	15.67
Neither bad nor good		74	49.33	41	27.33	115	38.33
Good		40	26.67	76	50.67	116	38.67
Very good		9	6.00	13	8.67	22	7.33
**The Perception of Health** **WHOQOL-BREF**		**SG**		**CG**		**Total**	
		**n**	**%**	**n**	**%**	**n**	**%**
Very dissatisfied		1	0.67	0	0.00	1	0.34
Dissatisfied		39	26.00	31	20.67	70	23.33
Neither satisfied nor dissatisfied		90	60.00	78	52.00	168	56.00
Satisfied		17	11.33	41	27.33	58	19.33
Very satisfied		3	2.00	0	0.00	3	1.00
**SG**							
**Quality of Life—Domains** **WHOQOL-BREF**	**M**	**SD**	**Me**	**Min**	**Max**	**Q1**	**Q3**
Physical	11.49	1.78	11	9	17	10	13
Psychological	12.79	1.96	13	6	17	11	14
Social	12.42	1.66	12	7	16	12	13
Environmental	12.51	1.57	12	7	17	12	14
**CG**							
**Quality of Life—Domains** **WHOQOL-BREF**	**M**	**SD**	**Me**	**Min**	**Max**	**Q1**	**Q3**
Physical	12.69	2.05	13	8	17	10	14
Psychological	14.63	2.77	15	9	19	12	16
Social	12.75	2.03	13	8	16	12	15
Environmental	13.15	1.83	14	9	16	12	14.75

M—mean, SD—standard deviation, Me—median, Min—minimum, Max—maximum, Q1—quartile 1, Q3—quartile 3, SG—study group and CG—comparative group. Source: authors’ analysis.

**Table 7:** The results of the assessment of the areas of quality of life in SG and CG.

WHOQOL-BREF Categories		Group		*p*
		SG (N = 150)	CG (N = 150)	
Overall quality of life	M ± SD	3.21 ± 0.81	3.55 ± 0.83	*p* = 0.001 *
	Me	3	4	
	Q1–Q3	3–4	3–4	
Satisfaction with health	M ± SD	2.88 ± 0.68	3.07 ± 0.69	*p* = 0.012 *
	Me	3	3	
	Q1–Q3	2–3	3–4	
Physical domain	M ± SD	11.49 ± 1.78	12.69 ± 2.05	*p* < 0.001 *
	Me	11	13	
	Q1–Q3	10–13	10–14	
Psychological domain	M ± SD	12.79 ± 1.96	14.63 ± 2.77	*p* < 0.001 *
	Me	13	15	
	Q1–Q3	11–14	12–16	
Social domain	M ± SD	12.42 ± 1.66	12.75 ± 2.03	*p* = 0.024 *
	Me	12	13	
	Q1–Q3	12–13	12–15	
Environmental domain	M ± SD	12.51 ± 1.57	13.15 ± 1.83	*p* < 0.001 *
	Me	12	14	
	Q1–Q3	12–14	12–14.75	

M—mean, SD—standard deviation, Me—median, Q1—quartile 1, Q3—quartile 3, *p*—significance level, SG—study group and CG—comparative group. * Mann-Whitney U-test. Source: authors’ analysis.

**Table 8:** Relationship between quality of life outcomes and lower extremity health.

**WHOQOL-BREF Categories**	**Total ISK Score**
Overall quality of life	r = −0.518, *p* < 0.001
Satisfaction with health	r = −0.634, *p* < 0.001
Physical domain	r = −0.347, *p* < 0.001
Psychological domain	r = −0.356, *p* < 0.001
Social domain	r = −0.031, *p* = 0.708
Environmental domain	r = −0.303, *p* < 0.001
**WHOQOL-BREF Categories**	**Total WOMAC Score**
Overall quality of life	r = −0.547, *p* < 0.001
Satisfaction with health	r = −0.686, *p* < 0.001
Physical domain	r = −0.424, *p* < 0.001
Psychological domain	r = −0.379, *p* < 0.001
Social domain	r = 0.018, *p* = 0.827
Environmental domain	r = −0.222, *p* < 0.006
**WHOQOL-BREF Categories**	**Subscale—Pain** **WOMAC**
Overall quality of life	r = −0.601, *p* < 0.001
Satisfaction with health	r = −0.68, *p* < 0.001
Physical domain	r = −0.416, *p* < 0.001
Psychological domain	r = −0.302, *p* < 0.001
Social domain	r = −0.078, *p* = 0.343
Environmental domain	r = −0.165, *p* < 0.044
**WHOQOL-BREF Categories**	**Subscale—Physical Activity** **WOMAC**
Overall quality of life	r = −0.52, *p* < 0.001
Satisfaction with health	r = −0.654, *p* < 0.001
Physical domain	r = −0.438, *p* < 0.001
Psychological domain	r = −0.442, *p* < 0.001
Social domain	r = 0.046, *p* = 0.573
Environmental domain	r = −0.214, *p* < 0.0008
**WHOQOL-BREF Categories**	**Subscale—Stiffness** **WOMAC**
Overall quality of life	r = −0.36, *p* < 0.001
Satisfaction with health	r = −0.4, *p* < 0.001
Physical domain	r = −0.572, *p* < 0.001
Psychological domain	r = −0.391, *p* < 0.001
Social domain	r = 0.026, *p* = 0.749
Environmental domain	r = −0.473, *p* < 0.001

*p*—statistical value; r—Spearman’s rank correlation coefficient.

## Data Availability

Not applicable.
